# Effective Mosquito Repellents: Myrcene- and Cymene-Loaded Nanohydrogels against *Aedes aegypti*

**DOI:** 10.3390/pharmaceutics16081096

**Published:** 2024-08-21

**Authors:** Jonatas Lobato Duarte, Leonardo Delello Di Filippo, Tais de Cássia Ribeiro, Ana Carolina de Jesus Silva, Lorane Izabel da Silva Hage-Melim, Stéphane Duchon, David Carrasco, Mara Cristina Pinto, Vincent Corbel, Marlus Chorilli

**Affiliations:** 1 Department of Drugs and Medicines, School of Pharmaceutical Sciences, São Paulo State University (UNESP), Araraquara 14800-903, São Paulo, Brazil; jl.duarte@unesp.br (J.L.D.); leonardo.filippo@unesp.br (L.D.D.F.); tais.cassiia@gmail.com (T.d.C.R.); 2 Departamento de Ciências Biológicas e da Saúde, Universidade Federal do Amapá, Macapá 68903-419, Amapá, Brazil; caroldejesus.farmacia@gmail.com (A.C.d.J.S.); loranehage@gmail.com (L.I.d.S.H.-M.); 3 IRD, CNRS, University of Montpellier, MIVEGEC, 34000 Montpellier, France; stephane.duchon@ird.fr (S.D.); david.carrasco@ird.fr (D.C.); vincent.corbel@ird.fr (V.C.); 4 Departamento de Ciências Biológicas, Faculdade de Ciências Farmacêuticas, Universidade Estadual Paulista “Júlio de Mesquita Filho”, Araraquara 14800-060, São Paulo, Brazil; mara.pinto@unesp.br

**Keywords:** carboxymethylcellulose, myrcene, cymene, arboviruses, nanoemulsion

## Abstract

Aedes mosquito-borne diseases remain a significant global health threat, necessitating effective control strategies. This study introduces monoterpenes-based nanohydrogels for potential use as repellents against *Aedes aegypti*, the primary dengue vector worldwide. We formulated hydrogels using cymene- and myrcene-based nanoemulsions with different polymers: chitosan, carboxymethylcellulose (CMC), and carbopol^®^. Our evaluations of rheological, texture, and bioadhesive properties identified CMC hydrogel as the most promising gelling agent for topical application, exhibiting sustained monoterpene release over 12 h with low skin permeation and high retention in the stratum corneum. Myrcene-loaded CMC hydrogel achieved a 57% feeding deterrence compared to 47% with cymene hydrogel in the mosquito membrane-feeding model. Molecular docking studies revealed interactions between myrcene and an essential amino acid (Ile116) in the *Ae. aegypti* odorant-binding protein 22 (AeOBP22), corroborating its higher repellent efficacy. These findings suggest that myrcene-loaded CMC hydrogels offer a promising, minimally invasive strategy for personal protection against *Ae. aegypti* and warrant further investigation to optimize monoterpene concentrations for vector control.

## 1. Introduction

Mosquitoes of the genus *Aedes* are primary vectors for arboviral diseases such as dengue, Zika, and chikungunya, collectively affecting over 50 million people worldwide yearly. This alarming scenario has seen a 30-fold increase in incidence over the past 50 years, closely associated with expanding urban populations [1]. These diseases pose significant global public health challenges, driving the urgent need for effective mosquito control strategies [2]. 

Conventional insecticides have been widely employed in efforts to manage mosquito populations. However, the rapid evolution of insecticide resistance in *Aedes aegypti*, the principal vector of dengue, has emerged as a significant concern [3]. Consequently, there is a pressing need for innovative approaches to control mosquito-borne diseases, particularly those that offer protection during outdoor human activities.

Repellents for personal protection have proven to be a practical and effective measure against mosquito bites, significantly reducing the transmission of various arthropod-borne diseases [4]. Natural products with repellent properties have recently garnered attention due to their low toxicity and environmental safety, offering promising alternatives to conventional insecticides [5]. Among these, monoterpenes found in essential oils have shown promising insecticidal and repellent properties [6]. 

Essential oils such as cinnamon oil and clove oil have demonstrated protection times exceeding 100 min against mosquitoes and ticks when used in 10% lotion emulsions [7]. Additionally, geranyl acetate and nerolidol, both individually and in mixtures, have shown nearly 100% protection rates with up to 3 h of efficacy, making them comparable to DEET [8]. The essential oil from *Croton tetradenius* leaves, rich in compounds like camphor, p-cymene, α-terpinene, and γ-terpinene, exhibited significant repellent activity with up to 100% blood-feeding inhibition at higher concentrations [9]. These studies highlight the potential of essential oils and their components, making them promising active ingredients for mosquito repellent formulations. However, rapid evaporation often limits their effectiveness when applied to the skin, resulting in short protection times [10]. 

Encapsulation of monoterpenes in nanotechnology-based formulations has enhanced their efficacy, prolonged their repellent action, and reduced skin permeation, thereby increasing safety [4,11]. Nanoemulsions offer a promising solution due to their small droplet size, extensive surface area, and heightened kinetic stability, contributing to improved solubilization and sustained release of active compounds [12]. Nanoemulsions of active compounds such as cinnamaldehyde, citral, and terpinen-4-ol have been particularly effective, significantly extending protection times in arm-in-cage assays compared to their non-nanoemulsified counterparts [13].

This study explores the potential of monoterpenes-based nanohydrogels as repellents against *Aedes aegypti*. We focused on formulating hydrogels using cymene and myrcene-based nanoemulsions incorporated with different polymers: chitosan, carboxymethylcellulose (CMC), and carbopol. We aim to identify the most effective formulation for sustained repellent action with minimal skin permeation by evaluating their rheological, texture, and bioadhesive properties. Furthermore, we investigated the repellent efficacy of these formulations and conducted molecular docking studies to elucidate their interactions with *Aedes aegypti* odorant-binding protein. Our findings offer insights into developing innovative, natural-repellent strategies to mitigate the impact of mosquito-borne diseases.

## 2. Materials and Methods

### 2.1. Nanoemulsion Preparation

The nanoemulsions (Cym-NE and Myr-NE) were prepared using a low-energy method described by Duarte et al. [10]. In brief, the oil phase, consisting of the terpene (p-cymene or myrcene) at 5% *w*/*w*, was combined with surfactants (Span^®^ 80 and Tween^®^ 20) at 5% *w*/*w* under magnetic stirring. After thorough homogenization, the aqueous phase (90% *w*/*w*) was gradually added dropwise. The resulting nanoemulsions exhibited a droplet size of approximately 120 nm and a uniform particle size distribution. These nanoemulsions demonstrated good colloidal stability, remaining stable for 90 days.

### 2.2. Preparation of the Hydrogel

Nanoemulsion-based hydrogels were prepared by dispersing 3% (*w*/*v*) of three different gelling polymers: Carbopol^®^ Ultrez 10 NF (Lubrizol, Wickliffe, OH, USA), low molecular weight chitosan (Sigma Aldrich St. Louis, MO, USA), and carboxymethylcellulose (CMC) (Synth, Sao Paulo, Brazil) into the previously obtained nanoemulsions under magnetic stirring. These polymers were selected based on literature recommendations [14,15]. To enhance bioadhesiveness, 0.5% Noveon^®^ Polycarbophil was added to all formulations [16]. The hydrogels were stored at room temperature for 24 h to ensure complete polymer swelling. The final concentration of active ingredient in the hydrogels was 500 mg/g of formulation.

### 2.3. Characterization of the Hydrogels

#### 2.3.1. Flow Rheology

Flow rheology experiments were conducted in a Discovery Hybrid HR-1 rheometer (TA Instruments, Newcastle, DE, USA) with cone-plate geometry (40 mm, 2°). Measurements were performed at 32.0 ± 0.1 °C using a shear rate of 0.1–100 s^−1^ for 120 s for the upward curve and 100–0 s^−1^ for 120 s for the descending curve. The flow behavior (n) and consistency index (k) were calculated using Equation (1):

(1) $$T=k\times \gamma^{n}$$

where τ—shear rate, k—consistency index, γ—shear stress, and n—flow behavior.

A Newtonian fluid is characterized by n = 1. A fluid exhibits dilatant behavior when n > 1 and pseudoplastic behavior when 0 < n < [17].

#### 2.3.2. Oscillatory Rheology

For the oscillatory analyses, the linear viscoelastic region (LVR) was determined using an amplitude range of 0–50 Pa and a frequency of 1 Hz. An amplitude of 1 Pa was defined within the LVR, and a frequency range of 0–10 Hz was used to determine the elastic (G′) and viscous (G″) moduli.

#### 2.3.3. Texture Profile Analysis (TPA)

The texture of the hydrogels was assessed using a TA-XTplus texture analyzer (Stable Micro Systems, Goldaming, United Kingdom). Seven grams of each hydrogel were placed in 50 mL conical tubes (Falcon, BD, Franklin Lakes, NJ, USA), centrifuged at 4000 rpm for 5 min to remove air bubbles, and left undisturbed for 24 h. The tubes were then subjected to uniaxial compression at 0.5 mm·s^−1^ to a depth of 10 mm, followed by a return to the sample surface at the same speed. A second compression was performed after a 5 s rest. All analyses were conducted in triplicate at 32 °C.

#### 2.3.4. Determination of the Hydrogel’s Bioadhesion

Bioadhesion was assessed using dermatomized pig ear skin. The skin was cleaned, trichotomized, and separated from the cartilage. A 400 μm stratum corneum layer and the epidermis/dermis were isolated using a dermatometer (Nouvag TCM 300, Goldach, Switzerland). A TA-Xtplus texture analyzer (Stable Micro Systems, England) with a cylindrical probe (10 mm ∅) was used to measure the force required to detach the skin from the hydrogel. The probe was lowered at 1 mm·s^−1^ until the skin touched the sample and penetrated to a depth of 1 mm. After 60 s of contact, the probe ascended at 0.5 mm·s^−1^ until the sample detached. All analyses were conducted in triplicate.

#### 2.3.5. In Vitro Release of Cymene and Myrcene from Hydrogels

In vitro release assays were performed under sink conditions using modified Franz cells in a Microette apparatus (Hanson Research, Thousand Oaks, CA, USA) with polyethersulfone membranes (Sigma-Aldrich, St. Louis, MO, USA). The receptor compartment was filled with 7.0 mL of 0.1 M phosphate buffer:ethanol (50:50) solution at pH 7.4, maintained at 37 ± 2 °C, and stirred at 300 rpm. Release was measured at intervals of 30 min, 1, 2, 4, 6, 8, 10, and 12 h, with compounds quantified by HPLC [18].

#### 2.3.6. In Vitro Skin Permeation and Retention of Cymene and Myrcene from Hydrogels

In vitro skin permeation and retention assays were conducted under sink conditions using Franz cells with a diffusion area of 1.77 cm^2^ in Microette equipment (Hanson Research, Thousand Oaks, CA, USA). The dermatomized porcine ear skin was exposed to the formulations for 8 h, employing the same experimental conditions as the in vitro skin permeation study. Following the experiment, the skin surfaces were thoroughly washed with distilled water to remove excess formulation and then placed in watch glasses to prevent drug loss. The stratum corneum was removed using the tape stripping technique, involving 16 adhesive tapes (Scotch^®^ 750 3M, Diadema, Brazil), with the initial tape discarded [19]. The tape strips were transferred to a test tube containing 5 mL of acetonitrile, vortexed for 1 min, and subjected to an ultrasound bath for 15 min to extract monoterpenes completely. The resulting solution was filtered through a 0.22 μm membrane (Merck^®^, Darmstadt, Germany) and injected into HPLC for quantifying cymene and myrcene [18].

After stratum corneum removal, the remaining skin (viable epidermis + dermis) was crushed with scissors. The fragments were placed in centrifuge tubes containing 5 mL of acetonitrile, stirred for 2 min, homogenized with a Turrax^®^ homogenizer for 1 min, and then subjected to an ultrasonic bath for 30 min. The solution was finally filtered through a 0.22 μm membrane (Merck^®^, Darmstadt, Germany), and cymene and myrcene were extracted and quantified using HPLC [18].

### 2.4. Membrane Feeding Assay in Mosquitoes

Adult females of *Aedes aegypti* (Bora strain) were used for the behavioral experiments. The membrane feeding assay (MFA) was used to test these hydrogels’ repellent effect more realistically [20,21]. Seven female mosquitoes were put in a paper cup covered with a mosquito net to prevent them from escaping, in which we offered them a blood meal. An artificial feeder was filled with rabbit blood at circa 37 °C, maintained by a circulating water bath. A porcine intestine was used as a membrane through which mosquitoes can bite and take their blood meal. Compounds of interest were applied on the exterior side of the membrane. 

The following treatments were applied: (i) control group without hydrogel; (ii) Cym-NE-hydrogel; (iii) Myr-NE-hydrogel. The mosquitoes were allowed to feed for 10 min and then visually inspected to check whether they were fed. The experiments were carried out in triplicate, with seven mosquitoes in each test. 

### 2.5. Molecular Docking of Cymene and Myrcene

To identify putative target receptors for cymene and myrcene, we conducted molecular docking to predict the binding affinity of each terpene with the OBP receptor. Two steps were performed before the molecular docking simulation: ligand optimization and redocking. The first, with the aid of the HyperChem program, through the use of the semi-empirical method Recife Model 1 (RM1) [22], aimed to optimize the energy and geometry of the ligands cymene (ICD: 7463), myrcene (ICD: 31253), and DEET (ICD: 4284), the latter being the reference molecule. 

Redocking, performed using the GOLD 2020.1 (Genetic Optimization for Ligand Docking) program, which is based on the genetic algorithm, serves to validate the program’s predictive ability to identify the active site of the macromolecule, where the ligand is in a favorable orientation in three-dimensional space [23,24]. It provides the source coordinate, the RMSD (Root Mean Square Derivation) value, and the validation radius. To this end, the crystallographic structure of the Ae. Aegypti odorant-binding protein 22 (OBP22), previously found to be associated with repellent’s binding, was obtained through the PDB (Protein Data Bank) database [25].

After the target was validated and verified through the RMSD value, molecular docking was performed to evaluate the interactions and types of interactions of the best ligand poses at the protein’s active site.

## 3. Results and Discussion

**Figure 1:**
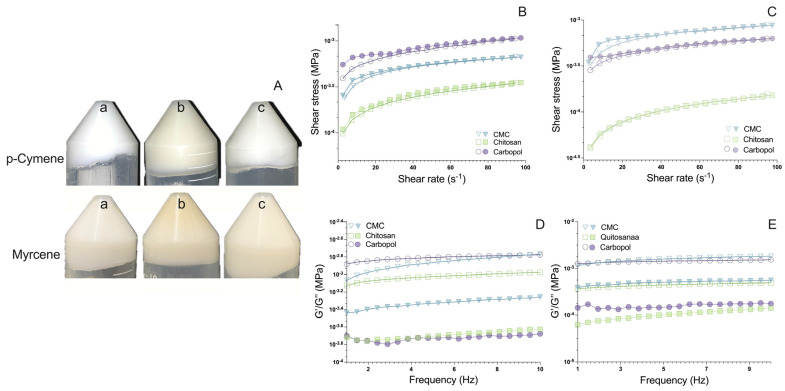
(**A**) Macroscopic aspect of the monoterpene-based nanohydrogels: a—Carbopol^®^ hydrogels (3% *w*/*w*), b—chitosan nanohydrogel (3% *w*/*w*), and c—CMC nanohydrogel (3% *w*/*w*); flow rheology of (**B**) Hydrogels containing Cym-NE; (**C**) hydrogels containing Myr-NE. Filled symbols indicate the ascending curves, empty symbols indicate the descending curves, and oscillatory rheology of the hydrogels containing the NETs. (**D**) Hydrogels containing Cim-NE and (**E**) hydrogels containing Mir-NE. Filled symbols indicate the G′ modulus, and empty symbols indicate the G″ modulus.

The incorporation of nanoemulsions into hydrogels requires polymers that meet several key criteria. Firstly, the polymers must have a good affinity for both the aqueous phase and surfactants in the nanoemulsion to ensure stable dispersion and prevent phase separation. Additionally, they must exhibit appropriate gelation properties to form a stable and homogeneous gel network that maintains the structural integrity of the hydrogel. The polymers should also provide suitable viscosity and rheological behavior, ensuring ease of application and good skin adhesion, particularly for topical formulations. Furthermore, bioadhesiveness is essential, allowing the hydrogel to adhere effectively to the skin, enhancing the efficacy of the active compounds. Finally, compatibility with the active ingredients in the nanoemulsion is crucial to maintain the stability and effectiveness of the formulation. In our study, Carbopol^®^, low molecular weight chitosan, and carboxymethylcellulose (CMC) were selected based on these requirements, as supported by the literature [26,27,28,29]. The hydrogels obtained in this study showed adequate macroscopic characteristics. Those obtained with Carbopol and CMC were white, while the chitosan hydrogel was yellow (Figure 1A). All hydrogels showed a homogeneous appearance and monoterpene-characteristic odor.

The rheological analysis provided insights into the mechanical behavior of the hydrogels. The ones formulated with chitosan demonstrated the lowest viscosity and exhibited no hysteresis area, indicating an immediate recovery of their internal structure post-shear application (Figure 1B,C). Conversely, hydrogels containing Carbopol^®^ and carboxymethylcellulose (CMC) showed thixotropic behavior, a desirable characteristic for topical applications due to its facilitation of spreadability and ease of removal from packaging [30,31]

All hydrogels exhibited pseudoplastic behavior (n < 1), which is advantageous for formulations intended for skin application (Table 1). The CMC hydrogel, particularly the myrcene-loaded one, exhibited the highest consistency index, indicating superior viscosity and structural integrity. This finding aligns with CMC’s known properties, forming a robust rheological matrix [32].

Oscillatory rheology results showed that all hydrogels were predominantly elastic (G′ > G″) (Figure 1D,E), which is beneficial for maintaining their shape and adherence on the skin [33,34]. The CMC hydrogels exhibited the highest loss modulus (G″), suggesting they have a significant energy absorption capacity, enhancing user comfort during application. This property can be attributed to the CMC matrix’s high viscosity and solid-like characteristics [17,33].

### 3.1. Texture Profile of Hydrogels

Texture Profile Analysis (TPA) highlighted significant mechanical property differences among the hydrogels. Hydrogels containing Carbopol^®^ and cymene nanoemulsions (Cim-NE) displayed the highest hardness, compressibility, adhesion, and cohesion values, likely due to the high viscosity imparted by Carbopol^®^ (Table 2). In contrast, myrcene-loaded hydrogels showed less variation across different polymers, suggesting that myrcene’s characteristics influence the mechanical properties less than cymene. The results indicate that carbopol is an excellent choice when high viscosity and robust mechanical properties are desired. In contrast, other polymers may have less impact on myrcene-containing formulations. These findings are significant for developing hydrogel formulations for pharmaceutical and cosmetic applications, where mechanical properties are crucial for both efficacy and user experience.

### 3.2. In Vitro Bioadhesion of Hydrogels

For topical products, assessing bioadhesion is a critical parameter in formulation development, as adhesion significantly impacts the effectiveness of these applications. Bioadhesion tests indicated that CMC and Carbopol hydrogels had superior bioadhesion compared to chitosan-based hydrogels (Table 2). High bioadhesion ensures prolonged skin contact [34,35], which is crucial for effective repellent formulations. Consequently, CMC was chosen for further studies due to its optimal combination of bioadhesion, viscosity, and mechanical properties.

### 3.3. In Vitro Release Test of Monoterpenes from Hydrogels

**Figure 2:**
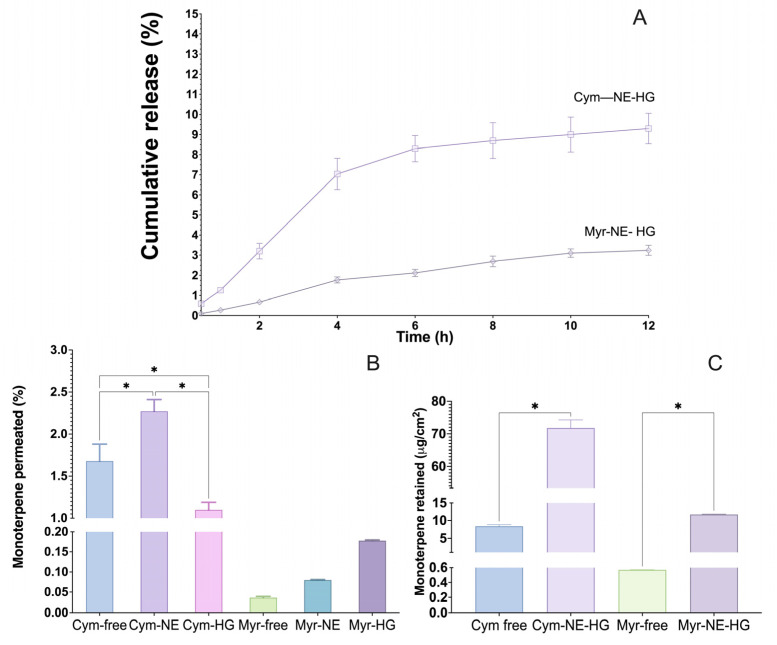
(**A**) In vitro release profile of the hydrogels containing the NETs, (**B**) in vitro permeation, and (**C**) retention profile of free monoterpenes, NETs, and hydrogels containing NETs after 8 h of experiment. The data are expressed as mean ± SD, one way ANOVA, * *p* ≤ 0.05.

The in vitro release profiles of cymene and myrcene from the CMC-based hydrogels were investigated over 12 h. The initial release of monoterpenes showed significant differences between cymene and myrcene. After 30 min, the cymene hydrogel released 0.59% of the compound, while the myrcene hydrogel released only 0.1% (Figure 2A). This indicates that cymene’s initial release was faster than myrcene’s. The results suggested that the release of monoterpenes from the hydrogels based on CMC was significantly lower than the release of the nanoemulsions [36]. This observation is consistent with previous studies that have reported the ability of nanoemulsions to provide a faster and more effective release of active compounds compared to hydrogel-based drug delivery systems [37,38].

The sustained release profile was evident as the monoterpenes released gradually increased. By the end of the 12 h, the cymene hydrogel released 9.3% of the compound, whereas the myrcene hydrogel released 3.24% (Figure 2A). This suggests that the CMC hydrogel matrix effectively controlled the release of the monoterpenes, with myrcene exhibiting a more controlled and sustained release than cymene. These findings are consistent with previous studies reporting the ability of hydrogels to provide a gradual and prolonged release of encapsulated compounds [37,38].

The slower release of myrcene can be attributed to its lower solubility and higher affinity to the CMC hydrogel matrix, which may result in stronger interactions and more significant retention within the hydrogel network [39]. This controlled release profile is advantageous for topical applications, ensuring a prolonged repellent effect on the skin.

### 3.4. In Vitro Permeation and Retention

After 8 h, different levels of monoterpene permeation were observed. The free cymene showed a substantial release, with 1.68 ± 0.2 detected in the permeation medium. On the other hand, 2.27 ± 0.14 of cymene from nanoemulsion reached the permeation medium. In the case of myrcene, after 8 h of permeation, the quantified amount of free myrcene was 0.036 ± 0.003. A slightly higher amount permeated was observed for the myrcene nanoemulsion, at 0.08%± 0.03. Regarding hydrogels, in the case of cymene, the monoterpenes permeated 1.1 ± 0.09, and with myrcene, 0.178 ± 0.001 (Figure 2B).

These results suggest that nanoemulsification may facilitate the permeation of monoterpenes, possibly due to its ability to improve the solubility of these compounds in aqueous systems. Due to the diminutive size of the droplets, nanoemulsification can penetrate further into the skin, thus increasing permeation compared to free terpenes [40,41].

The permeation and retention of cymene and myrcene from the hydrogels were evaluated using porcine ear skin in Franz diffusion cells. The permeation studies demonstrated that the nanoemulsified monoterpenes had low skin permeation rates but high retention in the stratum corneum, which is crucial for their effectiveness as repellents.

After 8 h, the amount of free cymene retained was 11 ± 0.09 μg/cm^2^, while the free myrcene permeated was 0.178 ± 0.001 μg/cm^2^ (Figure 2C). These results indicate that both monoterpenes had minimal permeation through the skin, with myrcene showing significantly lower permeation than cymene (Figure 2C. The low permeation rates benefit repellent formulations as they minimize systemic absorption and potential toxicity [14,42,43].

Retention studies showed that the monoterpenes from hydrogels were predominantly retained in the stratum corneum. Cymene retention was 71.8 ± 2.5 μg/cm^2^, whereas myrcene retention was 117 ± 12 μg/cm^2^ (Figure 2C). The high retention of monoterpenes in the stratum corneum is advantageous as it ensures prolonged surface activity where mosquito contact occurs, enhancing the repellent effect.

The ability of the CMC hydrogel to retain monoterpenes within the skin’s outermost layer while limiting their permeation aligns with the desired characteristics for topical repellents. This ensures that the active compounds remain on the skin surface, providing effective repellent action while minimizing the risk of systemic exposure.

Due to their distinct physicochemical properties, cymene and myrcene differ in release and permeation. Myrcene’s higher affinity for the CMC matrix results in a more controlled release and greater retention, making it a promising candidate for developing long-lasting repellent formulations [39]. These findings align with previous studies that have shown the potential of using polymer-based hydrogels to enhance the efficacy and safety of topical applications [16,41].

Considering the use of these monoterpenes in repellent formulations, the ability of hydrogels to retain the compounds in the skin’s stratum corneum can be seen as an advantage. This means that even if the permeation to deeper layers is limited, the repellent action can be maintained on the skin’s surface, where insects usually make contact, making hydrogels suitable for topical applications by providing a more prolonged action on the layer of the skin. This understanding can guide the design and formulation of more effective repellents, thereby contributing to protection against disease-carrying insects.

### 3.5. Repellent Efficacy of Nanoemulsions

The repellent efficacy of the nanohydrogels was evaluated using a membrane-feeding model with Aedes aegypti females. This assay objectively assesses the repellents’ effectiveness in deterring mosquitoes from blood-feeding.

**Figure 3:**
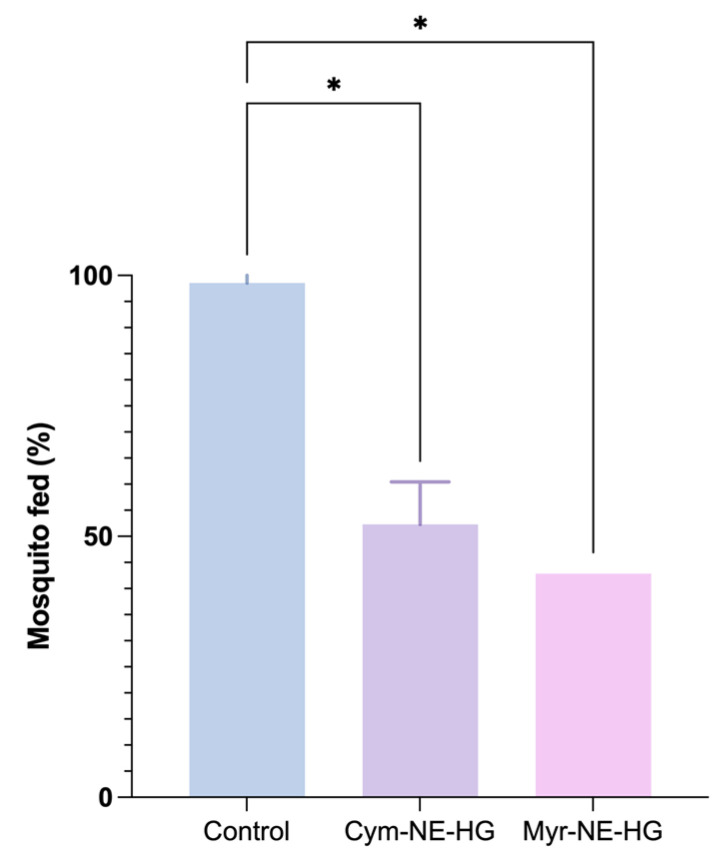
Percentage of mosquitoes fed with the control and the monoterpene hydrogels. One-way ANOVA, * *p* ≤ 0.05.

The control group, which contained no hydrogel, showed that 95% of the mosquitoes successfully fed on the artificial membrane, validating the assay setup (Figure 3). In contrast, monoterpenes-based nanohydrogels significantly reduced the mosquitoes’ feeding rate.

The myrcene-loaded CMC hydrogel exhibited the highest repellency, with 57% of the mosquitoes avoiding feeding. This performance was superior to the cymene-loaded CMC hydrogel, which deterred 47% of the mosquitoes from feeding. These results indicate that the myrcene formulation has a more substantial repellent effect than the cymene formulation.

The higher efficacy of the myrcene-loaded hydrogel is consistent with previous research emphasizing myrcene’s repellent properties against various insect species. Bedini et al. (2015) demonstrated myrcene’s repellent activity against *Rhyzopertha dominica* [44]. Similarly, Aguiar et al. (2015) reported that myrcene-rich *Siparuna guianensis* essential oil showed superior repellent activity against *Aedes aegypti* and *Culex quinquefasciatus* [45].

Similarly, cymene has also been shown to possess repellent properties, although to a lesser extent than myrcene. Choy et al. (2002) demonstrated that p-cymene exhibited repellent activity against *Culex pipiens* pallens, though it was less effective than DEET [46]. The results from our study confirm these findings, showing that cymene is effective but less potent than myrcene in repelling Aedes aegypti mosquitoes.

### 3.6. Molecular Docking

Molecular docking studies further supported the experimental findings by revealing significant interactions between cymene and myrcene with AeOBP22, but with different binding affinities and interaction profiles. The reference molecule DEET exhibited a gold score of 59.57, serving as a benchmark for comparing the monoterpenes (Table 3).

**Figure 4:**
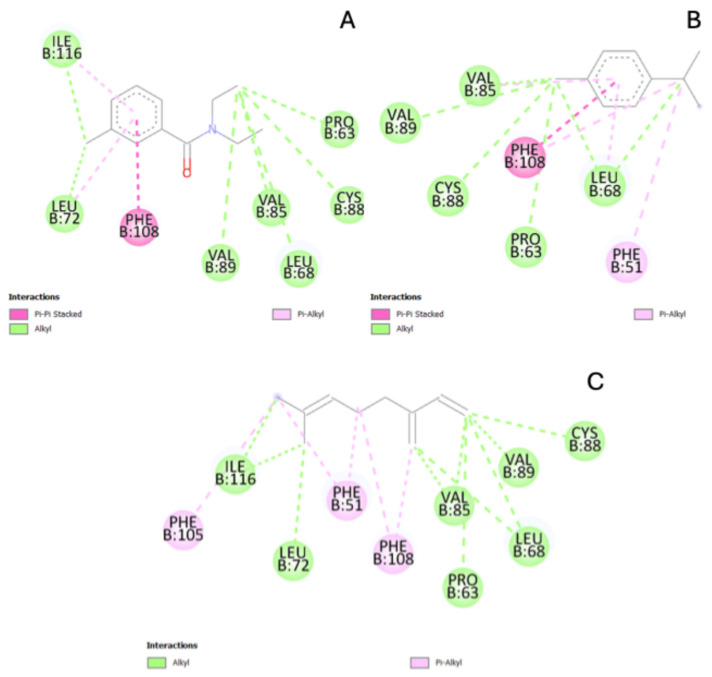
Molecular docking showing the interactions between (**A**) DEET, (**B**) cymene, and (**C**) myrcene with the AeOBP22 target.

Cymene displayed a gold score of 51.92, indicating a relatively strong binding affinity to AeOBP22, though lower than DEET. The interactions included π–π stacked interaction between cymene and Phe108, hydrophobic alkyl interactions with amino acids Pro63, Leu68, Val85, Cys88, and Val89, and π–alkyl interactions involving Phe51 and Phe108, enhancing the stability of cymene within the binding pocket (Figure 4B). These interactions suggest that cymene can effectively bind to AeOBP22, potentially interfering with the mosquito’s olfactory processes, though not as efficiently as DEET or myrcene.

Myrcene demonstrated a higher gold score of 52.55, reflecting a stronger binding affinity than cymene. The fundamental interactions included hydrophobic alkyl interactions involving Ile116, Leu72, Leu68, Val85, Pro63, Cys88, Val89, and Phe51, and, notably, π–alkyl interactions with Phe51, Phe105, and Phe108, which are crucial for stabilizing the ligand in the binding site (Figure 4C). The significant interaction between myrcene and Ile116, an essential amino acid in AeOBP22, underscores myrcene’s potential to disrupt the mosquito’s olfactory system more effectively than cymene. This higher binding affinity aligns with myrcene-loaded CMC hydrogels’ observed superior repellent efficacy in the membrane-feeding assay.

DEET, a widely used synthetic repellent, exhibited the highest binding affinity among the tested compounds, with a gold score of 59.57. Its interactions included multiple hydrophobic alkyl interactions with amino acids Phe108, Pro63, Leu68, Val85, Cys88, Leu72, and Ile116, alongside π–π stacked and π–alkyl interactions (Figure 4A). These interactions highlight DEET’s robust binding and repellent efficacy, serving as a benchmark for evaluating natural repellents like cymene and myrcene.

The molecular docking results explain how cymene and myrcene interact with AeOBP22, explaining their differing repellent efficacies. The stronger binding affinity of myrcene, particularly its interaction with Ile116, correlates with its higher repellent activity observed in the biological assays. These findings are consistent with the literature, where monoterpenes like myrcene have been shown to possess significant repellent properties against various insect species [44,45]. The molecular docking insights validate the potential of myrcene as a natural repellent, with its higher affinity for AeOBP22 disrupting the mosquito’s ability to detect human hosts effectively.

Furthermore, nanohydrogels enhance these monoterpenes’ delivery and sustained release, ensuring prolonged repellent action. The controlled release profile and low skin permeation rates observed in the in vitro studies complement the molecular docking results, supporting the practical application of myrcene-loaded CMC hydrogels as effective repellents.

The molecular docking studies elucidate how myrcene and cymene exert repellent effects through interactions with AeOBP22. Myrcene’s higher binding affinity and specific interactions with vital amino acids like Ile116 underscore its superior efficacy compared to cymene. These molecular insights, combined with the sustained release and high retention observed in the in vitro studies, highlight the potential of myrcene-loaded nanohydrogels as a promising natural alternative to synthetic repellents like DEET. Further research is warranted to optimize these formulations for real-world applications, offering a sustainable and effective solution for mosquito control [4,11].

## 4. Conclusions

This study demonstrated that myrcene and cymene-loaded nanohydrogels represent safe and effective natural repellents against *Aedes aegypti* mosquitoes. The CMC hydrogels exhibited excellent texture and adhesion profiles, sustained monoterpene release over 12 h, and low skin permeation with high retention in the stratum corneum. These characteristics are critical for ensuring prolonged repellent efficacy when applied topically. The myrcene-loaded CMC hydrogel showed superior performance, deterring 57% of mosquitoes from blood-feeding compared to 47% for the cymene-loaded hydrogel.

Molecular docking studies provided a mechanistic understanding of myrcene’s higher efficacy. They revealed significant interactions with the essential amino acid Ile116 in the AeOBP22 protein, corroborating its stronger repellent activity. These interactions likely disrupt the mosquito’s olfactory system, enhancing the repellent effect.

Our findings suggest that myrcene- and cymene-loaded nanohydrogels, especially those formulated with CMC, offer a promising and minimally invasive alternative strategy for personal protection against Aedes aegypti. These natural repellents could reduce reliance on synthetic chemicals like DEET, providing a more sustainable and environmentally friendly solution for mosquito-borne disease prevention. Further investigations are needed to optimize the concentrations and formulations of these nanohydrogels for real-world applications in vector control.

Developing these innovative formulations marks a significant step towards improving public health strategies against mosquito-borne diseases. Future research should focus on in vivo efficacy testing, long-term safety assessments, and the potential integration of these natural repellents into existing mosquito control programs to fully realize their benefits in protecting human populations from arboviral infections.

## Figures and Tables

**Table 1:** Consistency index (K) and flow behavior (n) for the hydrogels containing the NETs.

	Consistency Index (k) (Pa·s)	Flow Behavior Index (n)	R^2^
C-carbopol	389.79	0.214	0.93
C-Chitosan	77.3	0.332	0.99
C-CMC	215.16	0.246	0.99
M-Carbopol	258.89	0.187	0.92
M-Chitosan	23.32	0.406	0.99
M-CMC	328.16	0.208	0.97

C—cymene; M—myrcene; CMC—carboximetilcelulose.

**Table 2:** Mechanical parameters of hydrogels containing NETs.

Formulation	Hardness (g)	Compressibility (g⋅s)	Adhesivity (g⋅s)	Cohesion	Bioadhesion (g⋅s)
C-Carbopol	40.17 ± 3.50 ^A^	290.70 ± 21.18 ^A^	142.55 ± 29.78 ^A^	217.99 ± 5.67 ^A^	27.72 ± 1.87 ^A^
C-Chitosan	10.16 ± 1.34 ^B^	70.31 ± 9.47 ^B^	58.12 ± 7.78 ^B^	58.59 ± 7.46 ^B^	6.06 ± 1.39 ^B^
C-CMC	22.10 ± 2.17 ^C^	153.78 ± 13.70 ^C^	85.25 ± 5.86 ^C^	106.63 ± 7.95 ^C^	31.73 ± 2.54 ^C^
M-Carbopol	27.37 ± 0.79 ^A^	203.96 ± 25.31 ^A^	112.22 ± 12.86 ^A^	152.70 ± 10.54 ^A^	17.56 ± 4.10 ^A^
M-Chitosan	4.71 ± 0.17 ^B^	29.19 ± 0.83 ^B^	20.54 ± 0.80 ^B^	24.51 ± 0.748 ^B^	7.5 ± 0.18 ^B^
M-CMC	30.44 ± 2.16 ^C^	205.06 ± 15.52 ^A^	127.30 ± 9.75 ^C^	151.47 ± 11.40 ^A^	39.64 ± 2.01 ^C^

C—cymene; M—myrcene; CMC—carboxymethylcellulose. Different letters indicate statistically significant differences, one-way ANOVA (*p* ≤ 0.05).

**Table 3:** Interactions and interactions between cymene ligands, myrcene, DEET, and the OBP22 target.

	AA	Atom	Interaction	Type	Distance	Score
DEET	PHE108	Ligand	Hydrophobic	Pi-pi Stacked	5.22	59.57
	PRO63	C1	Hydrophobic	Alkyl	4.43	
	LEU68	C1	Hydrophobic	Alkyl	4.86	
	VAL85	C1	Hydrophobic	Alkyl	4.49	
	CYS88	C1	Hydrophobic	Alkyl	4.74	
	VAL89	C1	Hydrophobic	Alkyl	4.71	
	LEU72	C14	Hydrophobic	Alkyl	4.20	
	ILE116	C14	Hydrophobic	Alkyl	4.26	
	LEU72	Ligand	Hydrophobic	Pi-Alkyl	5.33	
	ILE116	Ligand	Hydrophobic	Pi-Alkyl	4.22	
Cymene	PHE108	C1	Hydrophobic	Pi-Pi Stacked	4.84	51.92
	LEU68	C10	Hydrophobic	Alkyl	5.36	
	PRO63	C10	Hydrophobic	Alkyl	4.62	
	LEU68	C10	Hydrophobic	Alkyl	4.95	
	VAL85	C10	Hydrophobic	Alkyl	4.33	
	CYS88	C10	Hydrophobic	Alkyl	4.57	
	VAL89	C10	Hydrophobic	Alkyl	4.67	
	LEU68	Ligand	Hydrophobic	Alkyl	4.29	
	PHE51	C1	Hydrophobic	Pi-alkyl	4.80	
	PHE108	C1	Hydrophobic	Pi-alkyl	5.18	
	VAL85	Ligand	Hydrophobic	Pi-alkyl	5.27	
Myrcene	ILE116	C6	Hydrophobic	Alkyl	3.90	52.55
	LEU72	C7	Hydrophobic	Alkyl	4.64	
	ILE116	C7	Hydrophobic	Alkyl	3.82	
	LEU68	C9	Hydrophobic	Alkyl	4.32	
	VAL85	C10	Hydrophobic	Alkyl	4.05	
	PRO63	C10	Hydrophobic	Alkyl	4.18	
	LEU68	C10	Hydrophobic	Alkyl	5.29	
	VAL85	C10	Hydrophobic	Alkyl	5.19	
	CYS88	C10	Hydrophobic	Alkyl	4.68	
	VAL89	C10	Hydrophobic	Alkyl	4.80	
	PHE51	Ligand	Hydrophobic	Pi-alkyl	5.09	
	PHE51	C6	Hydrophobic	Pi-alkyl	4.62	
	PHE105	C6	Hydrophobic	Pi-alkyl	4.71	
	PHE108	Ligand	Hydrophobic	Pi-alkyl	4.88	
	PHE108	C9	Hydrophobic	Pi-alkyl	3.95	

## Data Availability

The raw data supporting the conclusions of this article will be made available by the authors on request.
